# A virtual shopping test for realistic assessment of cognitive function

**DOI:** 10.1186/1743-0003-10-59

**Published:** 2013-06-18

**Authors:** Sayaka Okahashi, Keiko Seki, Akinori Nagano, Zhiwei Luo, Maki Kojima, Toshiko Futaki

**Affiliations:** 1 Division of Occupational Therapy, Department of Human Health Sciences, Graduate School of Medicine, Kyoto University, Kyoto, Japan; 2 Department of Computational Science, Graduate School of System Informatics, Kobe University, Kobe, Japan; 3 Department of Rehabilitation Sciences, Graduate School of Health Sciences, Kobe University, Kobe, Japan; 4 Department of Rehabilitation Medicine, Nishiyamato Rehabilitation Hospital, Nara, Japan

**Keywords:** Virtual reality, Cognitive function, Realistic assessment, Development, Brain injury

## Abstract

**Background:**

Cognitive dysfunction caused by brain injury often prevents a patient from achieving a healthy and high quality of life. By now, each cognitive function is assessed precisely by neuropsychological tests. However, it is also important to provide an overall assessment of the patients’ ability in their everyday life. We have developed a Virtual Shopping Test (VST) using virtual reality technology. The objective of this study was to clarify 1) the significance of VST by comparing VST with other conventional tests, 2) the applicability of VST to brain-damaged patients, and 3) the performance of VST in relation to age differences.

**Methods:**

The participants included 10 patients with brain damage, 10 age-matched healthy subjects for controls, 10 old healthy subjects, and 10 young healthy subjects. VST and neuropsychological tests/questionnaires about attention, memory and executive function were conducted on the patients, while VST and the Mini-Mental State Examination (MMSE) were conducted on the controls and healthy subjects. Within the VST, the participants were asked to buy four items in the virtual shopping mall quickly in a rational way. The score for evaluation included the number of items bought correctly, the number of times to refer to hints, the number of movements between shops, and the total time spent to complete the shopping.

**Results:**

Some variables on VST correlated with the scores of conventional assessment about attention and everyday memory. The mean number of times referring to hints and the mean number of movements were significantly larger for the patients with brain damage, and the mean total time was significantly longer for the patients than for the controls. In addition, the mean total time was significantly longer for the old than for the young.

**Conclusions:**

The results suggest that VST is able to evaluate the ability of attention and everyday memory in patients with brain damage. The time of VST is increased by age.

## Background

Cognitive function plays an important role in everyday life. Clinically, this function is recognized to have several aspects: attention, perception, memory, verbal function, executive function and so on. Basically, attention is the most fundamental one, followed by consciousness. In such a way, these functions form a hierarchical structure to realize the human’s cognitive function in their everyday life. Higher cognitive dysfunction caused by brain injury such as stroke and traumatic brain injury seriously influence the independent life for patients. Patients demonstrate difficulties in activities of daily living (ADL) and instrumental activities of daily living (IADL) [1,2].

Traditionally, medical staff and therapists use neuropsychological tests with pencil and paper for objective assessment of higher cognitive function. They usually use specific assessment for each cognitive aspect. For example, line cancellation test [3] is used as a test for spatial attention, and the Rey-Osterrieth Complex Figure Test [4] is used for visual memory. However, it is reported that results of neuropsychological tests sometimes disagree with the cognitive function level in real life of the patients [5-7]. It is difficult to evaluate the daily cognitive ability of people with cognitive dysfunction by only conventional neuropsychological tests. Therefore, in cognitive rehabilitation, before planning the detailed training program, it is important to understand that we should not only evaluate each cognitive function precisely, but also clarify the problems for the patients in their real life.

In this research, we propose a new approach to evaluate the people’s higher cognitive function using virtual reality (VR) technology so as to overcome the limitation of conventional types of assessments. VR technology provides one of the most advanced interactions between human and computers. In addition, this approach has the advantage of providing realistic scenario repeatedly, cost-effectively and safely for patients [8,9].

By now, there are already some reports proposing to use VR for the assessment [10-15]. Zhang et al. (2003) used a virtual kitchen for assessment of executive function in meal preparation task (e.g. a can of soup and a sandwich) [14]. In this task, the subject operated a personal computer (PC) by using a mouse and wearing a head-mounted display. They investigated the correlation among VR performance, actual kitchen performance, occupational therapy evaluation, and neuropsychological evaluation of people with brain injury. The virtual reality system showed adequate reliability and validity as a method of assessment. Knight et al. (2006) reported a virtual street for assessment of deficits in prospective memory following chronic traumatic brain injury (TBI) [12]. The virtual street was created by taking a series of 1500 photographs every few meters inside and outside of shops in the downtown shopping precinct of a real city in New Zealand. Subjects could move along the street by pressing buttons on the PC screen and complete ongoing and prospective memory tasks in the virtual street under conditions of high and low distraction. They were required to do ten errands with a checklist and respond to three targets that appeared repeatedly. Patients performed both tasks poorly compared with the controls as well as more affected by distractions.

These studies show assessment of realistic cognitive function using VR technology has significant possibility in future clinical rehabilitation. However, there were various problems when we applied these systems for patient with cognitive dysfunction in Japan. Firstly, some systems required the use a joystick which was difficult to operate for people who were unfamiliar with using a PC [10]. Secondly, the virtual environment and language used in these systems were foreign to the Japanese subjects. It was especially difficult for the elderly to overcome a cultural gap in addition to understanding unfamiliar scenery. Thirdly, it seems that the tasks were too complex. They required the subject to buy several items and to respond to three targets in one test session [11,12].

In our research, we developed a new VR system to evaluate cognitive function: the Virtual Shopping Test (VST). We emphasized the following three points during the system design: 1) the system should be easy for the beginners to operate, 2) it should represent everyday living environment in Japan, 3) the task levels should be appropriate for patients with cognitive dysfunction. With respect to the point 1), we used a touch screen as the interface instead of joystick. For point 2), we presented a virtual Japanese style shopping mall on the screen. In Japanese local shopping malls, there are shops on both sides of road, whose width is about five meters. The construction is relatively simple, which differs from Western style complex malls or hypermarkets. Most of shops are under small management, and a shop building is like a small house. They put up a signboard written in Japanese characters including Kanji. Usually, a shopping mall has from ten to thirty shops within walking distance. They are often located in front of a train station. Such kinds of shopping mall was much familiar by the Japanese elderly especially those who do not have foreign educations. For point 3), we arranged shopping tasks with four specific items and allowed users to refer to hints (e.g. shopping list and a view of the shopping bag) in case when they needed help.

The reason to introduce a shopping task in a virtual shopping mall is because we aimed to assess the cognitive ability in daily routine. In VST, subjects were required to memorize items to buy, to look for specific shops on a street, to choose items in a shop and perform the whole tasks smoothly. We assumed that subjects used ‘selective attention’ when they choose the target shop on a street and the correct item in a shop. They were required to use ‘memory’ when they memorize the four items on the shopping list and recall them at the appropriate time. The test also required the use of ‘executive function’ when the subjects completed the tasks quickly and efficiently by using the shortest way from start to goal.

Another feature of our VST is that a log file is recorded automatically. This file contains the important data during performing the tests (e.g. correct action and the time required to perform the test). Therefore, the examiner or clinician do not need specific skills to use VST, they can concentrate on the observation of the subjects’ behavior during the tests.

By our VST, we performed assessments of the realistic cognitive function for many subjects. Firstly, we tried to find out the significance of VST by comparing VST with conventional neuropsychological tests and questionnaires. We then tested the difference between brain-damaged patients and healthy subjects in a control to gauge the performance of VST. Finally, we investigated the age differences when using VST.

## Methods

### The Virtual Shopping Test

#### Experimental apparatus

**Figure 1:**
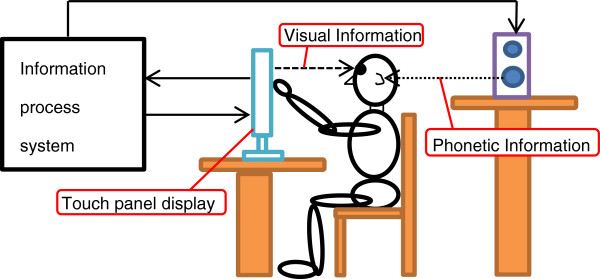
Composition of the experimental device.

**Figure 2:**
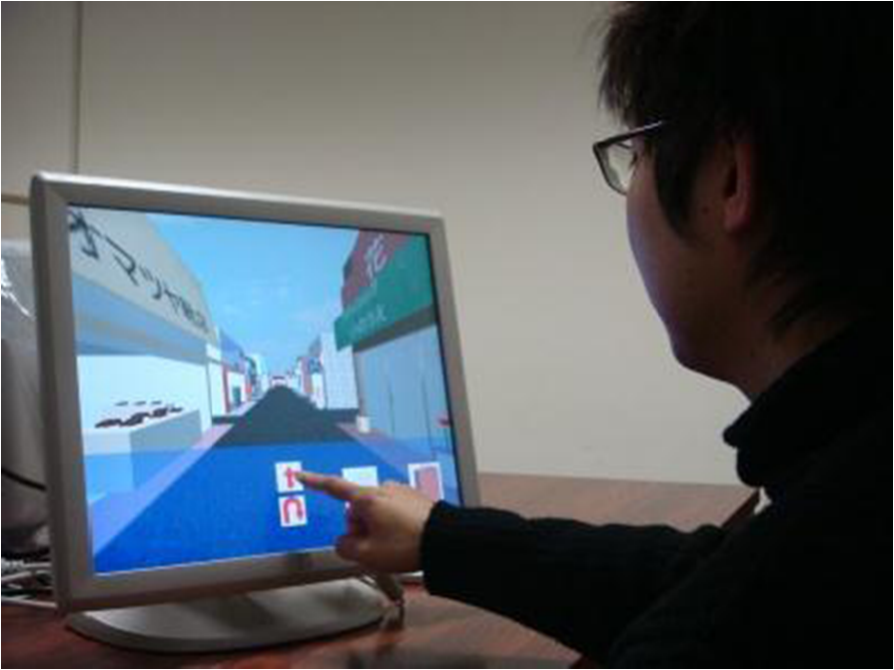
Scene of the experiment.

**Figure 3:**
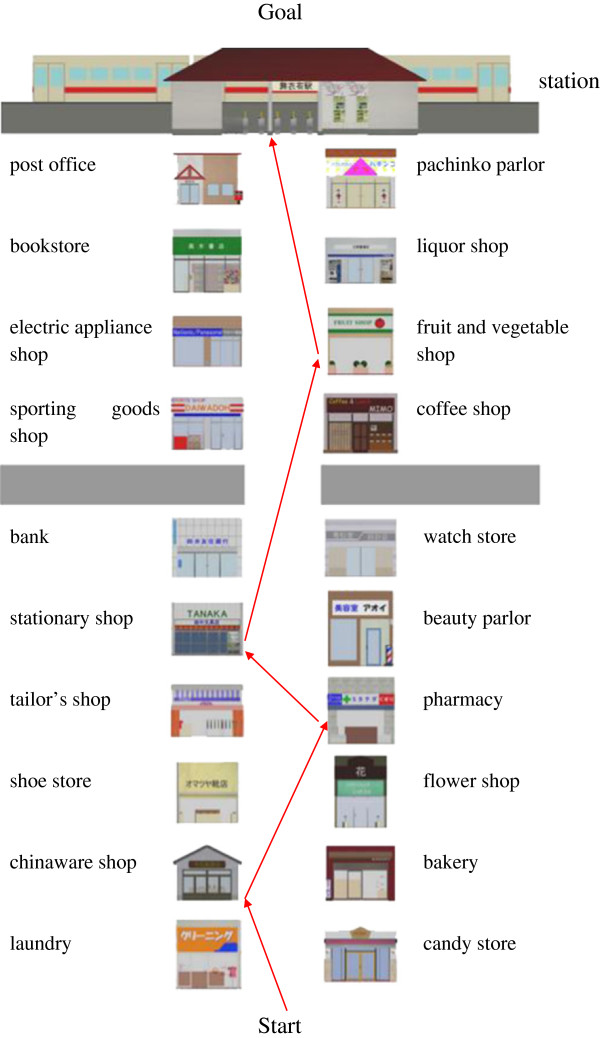
**Map of virtual shopping mall.** There are twenty shops and a station in the virtual Japanese shopping mall. The bottom is a start point, while the top is a goal. The red route shows the most efficient shopping order. We provided participants with an A4 sized sheet printed the map without the red route.

The hardware system included a personal computer and a touch screen (1928 L 19” LCD Desktop Touch monitor 5000series, Tyco Electronics, DE, USA). The virtual environment was developed with Metasequoia and Open GL. Figure 1 shows the overall setup of the experimental device, and Figure 2 shows the scene of the experiment. In this program, visual environment was made up of a Japanese shopping mall with 20 shops and a train station (Figure 3). An audio environment of natural sound of the shopping mall was also provided. By touching the bottom of the screen, users could move in the virtual shopping mall, entering a shop and buying an item.

#### Basic program and operation procedure

**Figure 4:**
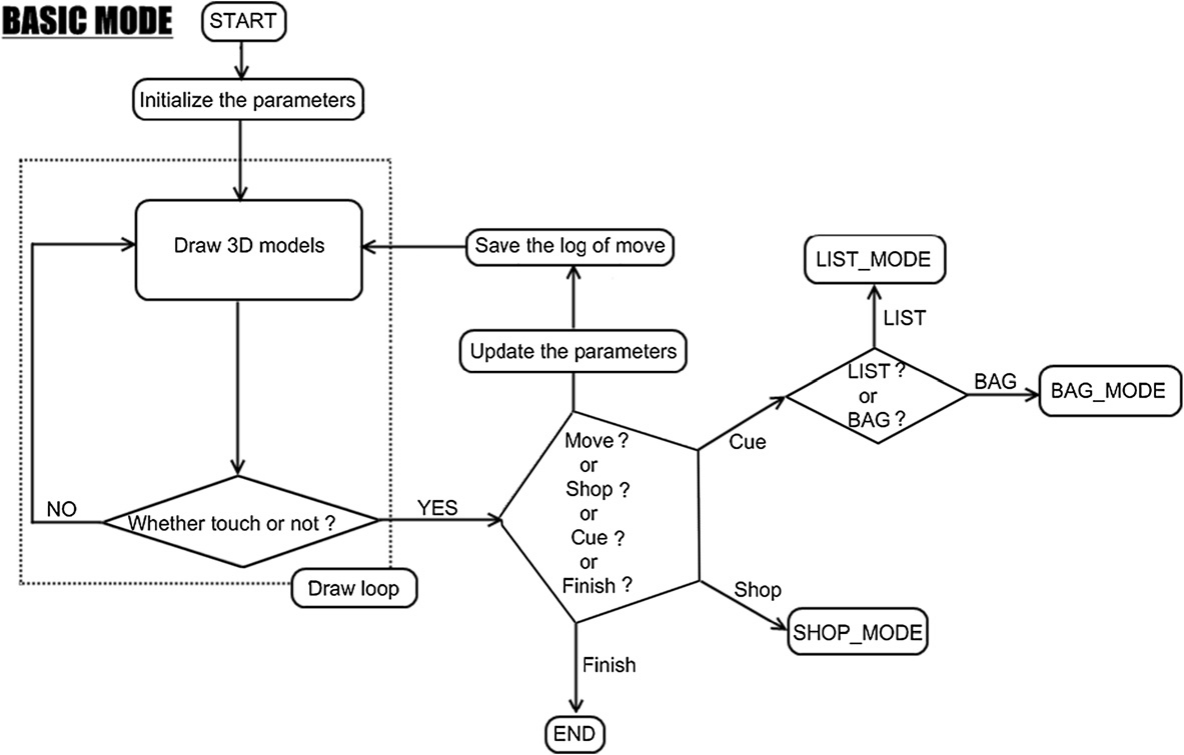
**BASIC MODE flow chart.** BASIC MODE is a fundamental mode of VST. In this mode, users can move in virtual shopping mall by touching the upper and lower arrow button. Users also can transfer to LIST/BAG MODE and SHOP MODE from this mode.

**Figure 5:**
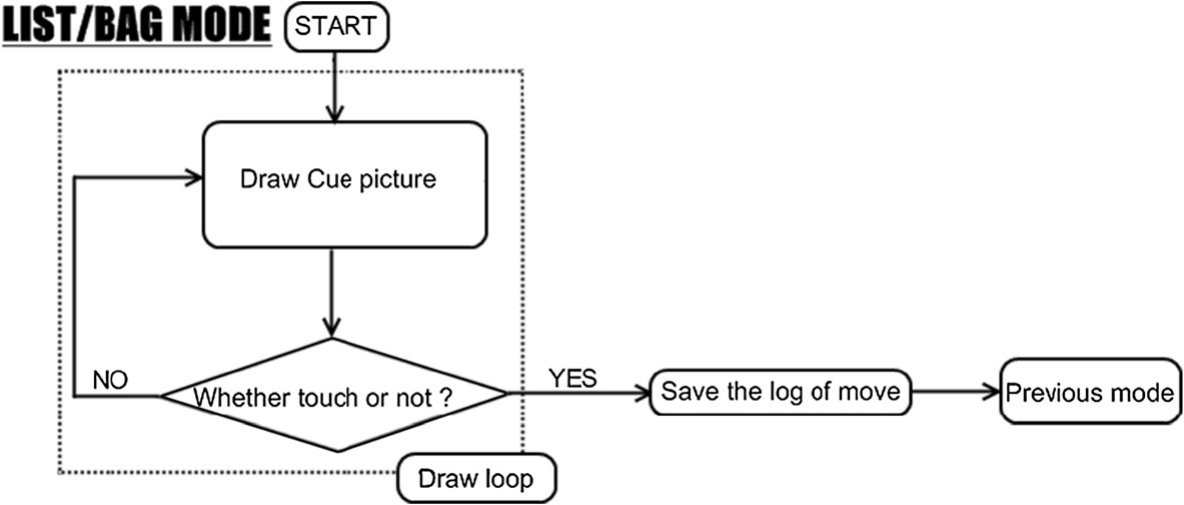
**LIST/BAG MODE flow chart.** If users forget the items in the shopping list or something they have bought already during VST performance, they can touch the list or bag button to transfer to LIST/BAG MODE.

**Figure 6:**
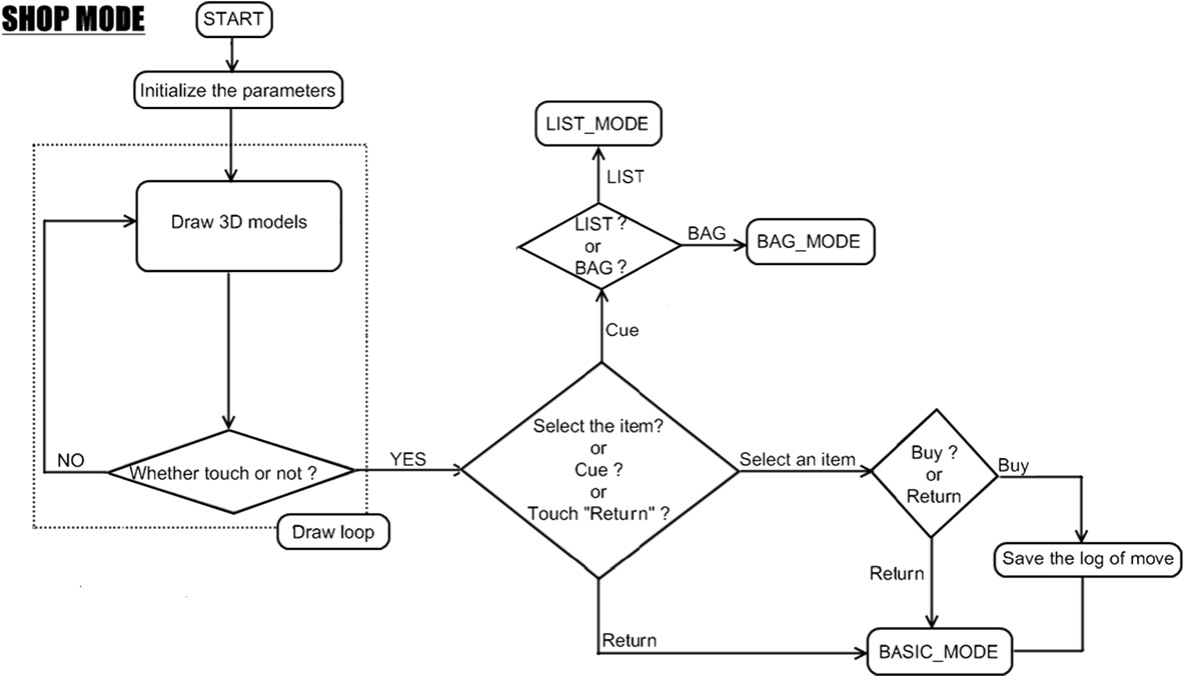
**SHOP MODE flow chart.** In this mode, users could enter a shop, select a shopping item and buy it. After finishing shopping, they would be transferred to BASIC MODE.

This system had 3 modes, namely BASIC MODE (Figure 4), LIST/BAG MODE (Figure 5), and SHOP MODE (Figure 6). These figures show a flow chart of the data processing for each mode.

**Figure 7:**
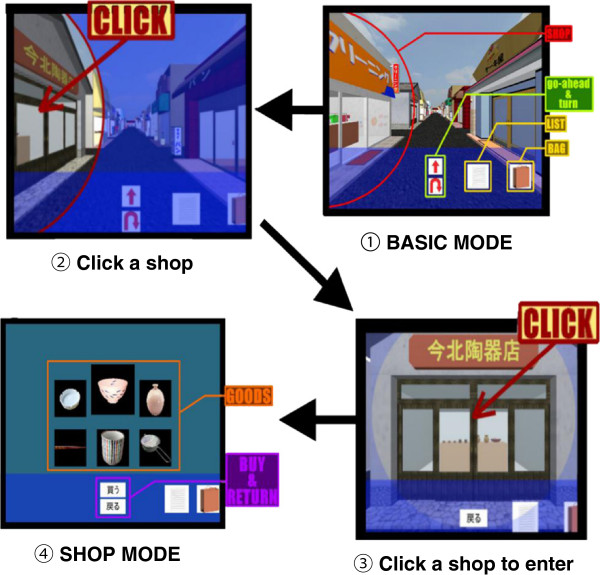
**Procedure of operation.** This figure shows transfer from BASIC MODE to SHOP MODE on the screen.

In BASIC MODE, there were 4 buttons on the bottom of the screen (Figure 7-①;). Two arrow buttons were provided to allow users to move and perform direction changes freely. By touching the upper arrow button, users can move forward to the next shop. By touching the lower button, users can turn 180 degrees at that point.

**Figure 8:**
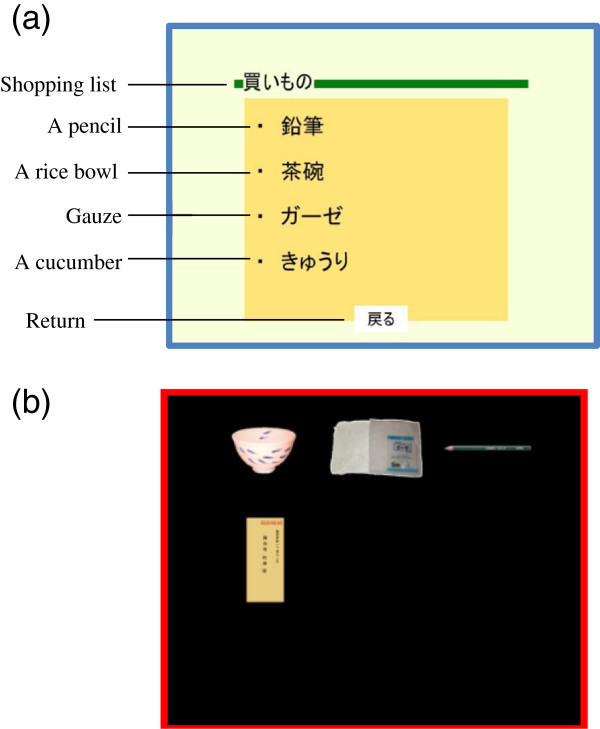
**Screens on LIST/BAG MODE.** (**a**) The screen on LIST MODE. On this mode, a shopping list with four Japanese words is displayed. (**b**) An example screen on BAG MODE. On this mode, items users have bought already are displayed.

Two hint buttons (e.g. List and Bag) were provided to allow users to view some hints during the shopping task. By touching the List button, the screen transfers to LIST MODE and all the names of the items on the shopping list are displayed (Figure 8a). By touching the Bag button, the screen transfers to BAG MODE and displays all the pictures of the items that were already bought (Figure 8b).

To enter a shop and buy an item, a user must approach the shop (Figure 7-①;). By touching the shop building, the screen was transferred to SHOP MODE and the full-screen picture of the shop was displayed (Figure 7-②, ③). By touching the shop picture again, users would be allowed to enter the shop (Figure 7-③, ④;). There were six shopping items displayed in every shop for sale. By touching one of the six items on the screen, the picture of the chosen item was enlarged. By touching the ‘buy’ button on the bottom of the screen, the item was placed into the shopping bag and the user could then exit the shop.

#### Task setting

We set a shopping task which asks users to buy four specific items. The user must search the shops that sell specific items and select the target item out of six items inside a shop. The four shopping items were “a pencil, a rice bowl, a piece of gauze, and a cucumber”. The items were selected from Japanese words with high imageability (above 5.6) based on the NTT Psycholinguistic Databases “Lexical properties of Japanese” [16]. Imageability is a semantic property that is defined as the ease and speed with which a target word evokes a corresponding mental image [17]. The words were also chosen from different word categories. The four shops that sold items (i.e. a stationary shop, a chinaware shop, a pharmacy, and a fruit and vegetable shop) were arranged such that two shops are located on each side of the street (Figure 3).

**Figure 9:**
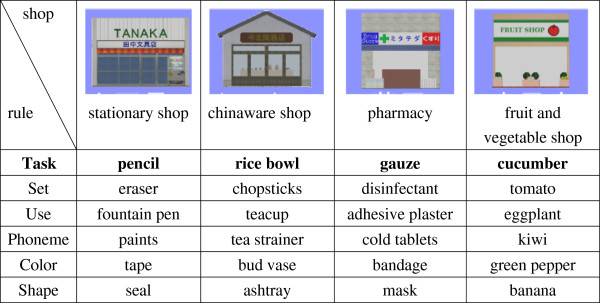
**Shopping options cluster.** This figure shows six shopping options in each target shop. Task means a correct item required to buy on VST.

**Figure 10:**
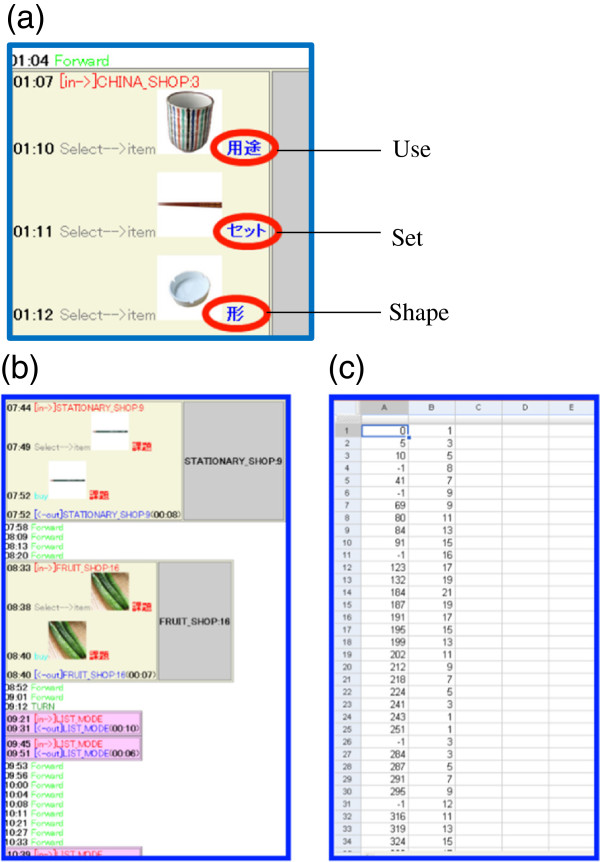
**Log files.** (**a**) The type of error recorded in a log file. (**b**) The HTML log form for reading. (**c**) The CSV log form for data management.

We arranged six shopping items in each shop with the following rule so that we could identify the reason for error when users made mistakes. The product cluster in each shop was shown in Figure 9. In a shop, there were 5 specific false items and a correct item. The 5 false items were either similar or associate with the correct item in some way. One false item was usually used with the correct item (Set). Another false item had the same usage as the correct item (Use). The 3^rd^ false item had the same initial sound in Japanese as the correct item (Phoneme). The 4^th^ item had the same color as the correct item (Color). The 5^th^ false item had the same shape as the correct item (Shape). These six items were arranged in a table of 2 rows by 3 columns on a screen (Figure 7-④;), and the place of correct item was set randomly. If a user bought a false item, the type of error was recorded with the picture in the HTML form in a log file (Figure 10a).

#### Data output

The operation of buttons during VST was recorded automatically, and outputted as a log file after finishing the test. There were two types of log file: the HTML form for reading (Figure 10b) and the CSV form for data analysis (Figure 10c).

### Experimental procedure

In the actual VST, the subjects were first asked to memorize four shopping items and were allowed to look at the shopping list for 10 seconds. If they failed the first recall test, they were allowed to memorize the items again in the same way. Secondly, subjects were allowed to plan their shopping routes that they thought would be the most efficient by filling in a blank map sheet with a pencil. They were then asked to finish buying the specific four items as quickly and efficiently as possible, while minimizing the use of hints as much as possible. Subjects were allowed to refer to their planning using the blank map on the table during the VST at any time.

Before the actual VST, the subjects were given a demonstration of the VR screen and a task to practice. The demonstration of the VR screen and instruction were provided to the subjects by an occupational therapist or a speech language pathologist according to a standardized manual. It took about thirty minutes including the demonstration for one session of VST.

#### Outcome variables

VST had ten outcome variables: Bag use, List use, Cue use, Forward movement, Reverse movement, Correct purchases, Total time, Time in shops, Time on road, and Mean time per shop. They could be calculated from the recording data.

Bag use, List use, Cue use, Forward movement and Reverse movement represent the number of times the subjects touched each button. For example, Bag use represents the number of times the subjects checked the items in the bag. List use represents the number of times the subjects referred to the shopping list. Cue use represents the total number of times the subjects checked the shopping list and bag. Forward movement represents the number of times that the subjects pressed upper arrow. Reverse movement represents the number of times the subjects pressed lower arrow. If subjects did not check the items in the bag or refer to the shopping list or reverse direction, then Bag use, List use, Cue use, Reverse movement would have a value of 0, which would represent the most efficient performance. If subjects completed the task by moving from start to goal with the shortest path (see the red route as shown in Figure 3), then Forward movement would have a value of 12, which would be the most efficient performance.

Correct purchases represents the number of items bought correctly. In VST, subjects were required to buy 4 specific items, and 4 would be the best score. The variable ranged from 0 to 4.

Total time, Time in shops, Time on road, and Mean time per shop represent the time required in each place. For example, Total time represents the total time spent to complete the whole shopping task. Time in shops represents the total time spent in each shop. Time on road represents the total time spent on road. Mean time per shop represents the average time spent in one shop. Regarding these variables, smaller number would represent higher performance.

#### Data collection

We have three objectives for this study: 1. to evaluate the significance of VST by comparing VST performance with conventional tests; 2. to test the applicability of VST to brain-damaged patients with normal subjects as a control group; and 3. to investigate the performance of VST in relation to age differences. For the first objective, participants came from patients with brain damage. While for the second objective, patients with brain damage were tested together with age-matched healthy subjects that were acted as control. In addition, old and young healthy subjects joined the experiment for the third objective.

### Participants

#### Patients and controls

Ten patients with cognitive function disorder (6 males, 4 females, mean age 43.5 ± 16.0 years, mean years of education 13.2 ± 1.7) and ten age-matched healthy subjects (4 males, 6 females, mean age 47.1 ± 20.1 years, mean years of education 14.1 ± 2.0) participated in this study.

For the patients, the participation criteria were as follows: 1) more than one year since brain damage onset, 2) between 20 and 69 years old, 3) cognitive ability to understand how to operate a touch screen, and 4) physical ability to reach and touch the screen by their uninvolved upper limbs. Demonstration to operate the screen were given to the participants and then they were given a practice session of operating the touch screen. The participants were then given a practice test to ensure that they had a good understanding to operate the VR system. If they failed the first test, demonstration and practice time would be provided to them again. They were given a maximum of 3 tries. The study exclusion criteria were as follows: i) severe aphasia, and ii) severe unilateral spatial neglect (USN).

A detailed description of the patient group is shown in Table 1. The patients included some forms of brain damage; five of them had stroke and another five had TBI experienced. They all had difficulties in their instrumental activity of daily living. The cognitive dysfunction was related to more than one aspects of ability (e.g. attention, memory, and executive function). They either lived at home with their family or received residential rehabilitation services at a prefectural rehabilitation center. The average time since onset was 52.3 ± 50.9 months, and the average Functional Independence Measure (FIM) score [18] was 112 ± 10.3.

**Table 1:** Patient description and cognitive assessment data

**Patient ID**	**1**	**2**	**3**	**4**	**5**	**6**	**7**	**8**	**9**	**10**
Age	32	36	61	51	57	62	59	28	22	27
Gender	M	M	M	M	M	F	F	M	F	F
Diagnosis	TBI	TBI	TBI	Stroke	Stroke	Stroke	Stroke	Stroke	TBI	TBI
Site of paralysis	right	left	none	none	left	left	left	left	bilateral	bilateral
Duration of disease (months)	143	144	21	45	25	16	23	17	18	71
General cognitive ability										
MMSE /30	27	22	26	30	29	20	28	30	26	25
Attention										
SDMT (%)	18	4	36	28	19	13	22	45	19	30
SRT: correct rate (%)	96.3	―	97.5	98.8	100	87.5	92.5	100	97.5	98.8
SRT: reaction time [ms]	295.4	―	312.1	315.9	373	551.7	296.7	273.8	391.5	374.6
Star Cancellation Task /54	54	54	52	54	54	54	48	54	54	54
Letter Cancellation Task /40	37	37	40	40	36	39	36	36	39	39
Memory										
RBMT: standard profile score /24	8	9	17	21	12	5	15	20	18	22
EMC /52	50	35	40	22	13	31	25	24	22	23
Executive function										
Zoo Map Test: the 1^st^ trial /8	−6	―	1	1	2	5	3	3	1	2
Zoo Map Test: the 2^nd^ trial /8	7	―	8	8	5	8	8	8	8	8
DEX /80	63	40	51	16	5	21	17	32	18	30

*M* male, *F* female, *TBI* traumatic brain injury.

For the controls, the inclusion criteria were as follows: 1) no history of neurological or psychiatric disorders, 2) between 20 to 69 years old. The subjects who scored less than 24 (cutoff score) on the Mini-Mental State Examination (MMSE) [19] were excluded.

#### Old and young healthy subjects

Ten old healthy subjects (1 males, 9 females, mean age 68.9 ± 3.9 years, mean years of education 13.0 ± 1.7) and ten young healthy subjects (5 males, 5 females, mean age 25.2 ± 3.0 years, mean years of education 14.9 ± 1.4) participated in this study. The inclusion criteria were as follows: 1) no history of neurological or psychiatric disorders, 2) between 60 and 79 years old for old group, between 20 and 29 years old for young group. The subjects who scored less than 24 points on MMSE were excluded.

There was no significant difference in average age and average years of education between the patients and controls. There was no significant difference in average years of education between the old and young healthy subjects. All participants received written and verbal information about the study and gave written informed consent. The protocol of the study was approved by Kobe University medical ethic committee.

#### Procedure

Patients were administered VST, seven conventional neuropsychological tests and two questionnaires, while the control and healthy subjects were administered VST and MMSE. General cognitive level was evaluated by MMSE. Attention was evaluated by symbol Digit Modalities Test (SDMT) [20] and Simple Reaction Time Task (SRT) [21]. Regarding visual inattention, the presence of USN was assessed by Star and Letter Cancellation Task [22,23]. Everyday memory was assessed by Rivermead Behavioural Memory Test (RBMT) [24,25] and Everyday Memory Checklist (EMC) [26]. Executive function was evaluated by Zoo Map Test and the Dysexecutive Questionnaire (DEX) [27]. A more detailed description of these tests is given in the following.

For the patients, the whole assessments were conducted on three separate date. All tests were finished within one month before and after VST execution. The total time was about five hours for one patient. For the controls and healthy subjects, the tests were conducted on one date within one hour.

#### Neuropsychological tests and questionnaires

MMSE: The MMSE was a short screening test grouped into 7 categories: orientation, registration, attention and concentration, recall, language, repetition, and visual construction. It was scored out of a possible 30 points. A higher score meant a better cognitive function while a lower score (< 24) indicates cognitive impairments [19].

SDMT: The SDMT was a visual test of processing speed. Subjects held a sheet that contained the numbers and symbols to be processed. The top row of stimuli included nine symbols, each of which was paired with a single digit in the key. The remainder of the page had a randomized sequence of these symbols, and the participant’s task was to respond orally with the digit associated with each of the symbols as quickly as possible. The SDMT lasted for 90 seconds and the completing rate was measured [20].

SRT: The SRT was one of the tasks of Continuous Performance Test (CPT) using a PC. The participant was required to press a specially marked key on the keyboard whenever a certain number appeared on the screen. During the test, the number appeared at intervals varying between 1000 and 2000 milliseconds (ms) and remained on the screen either until a response occurred or 1000 ms had elapsed. The total trial lasted approximately 3.3 min and consisted of 80 presentations of the target stimulus [21]. The correct rate and average reaction time were measured.

Star Cancellation Task: The Star Cancellation task from the Behavioural Inattention Test (BIT) consisted of 56 smaller stars interspersed with distractors: words, letters, stars. An A4 sized task sheet divided 27 right-sided and 27 left-sided stars on each half of the array. Two stars on this vertical line were not included in the analysis The patients’ task was to mark all of smaller stars [22,23].

Letter Cancellation Task: The Letter Cancellation Task from BIT consisted of 40 target letters arranged with other letter distracters. Japanese hiragana letters were printed in five lines of 34 items each, distributed equally on either side of the array on an A4 sized sheet. The patients’ task was to mark all of target letters [22,23].

RBMT: The RBMT was an ecologically valid, broad measure of impairment in everyday memory functioning. It was composed of 12 subtests which required the patient to remember a name and a last name, a short newspaper article, and a route. It also required the patient to recognize objects and faces; to remember to request a hidden belonging; to ask a question when an alarm rings; and to answer questions about temporal and spatial orientation. The standard profile score was the sum of all the subtest scores, which ranged from 0 to 24 [24,25].

EMC: The EMC consisted of 13 questions concerning memory problems in daily life [26]. Each answer was rated on a four-point scale (1–4), and the total score ranged from 13 to 52. Higher scores indicated more severe memory impairments.

Zoo Map Test: The Zoo Map Test from the Behavioural Assessment of the Dysexecutive Syndrome (BADS) was designed to assess the subjects' ability to formulate and implement a plan. Subjects were required to show how they would visit a series of designated locations on two drawn zoo maps without breaking a set of rules. In the first trial, they had to formulate the route (high-demand condition). In the second trial, they were simply required to follow written instructions (low-demand condition) [27,28]. In this study, the raw-score of each trial was adopted.

DEX: The DEX scale consisted of 20 questions concerning executive function problems in daily life including difficulties with attention, memory, information processing, behavioural control, emotion regulation and awareness. Each answer was rated on a five-point scale (0–4), and the total score ranged from 0 to 80. Higher scores indicated more severe impairments of executive function [27,28].

### Statistical analysis

Speaman’s correlations were performed to determine the association between VST and conventional cognitive assessments in the patient group. Beforehand, Speaman’s correlations were performed not only between each outcome variable in VST but also between each outcome variable in neuropsychological tests. If a highly significant statistical correlation in the specific variable group was observed, one variable of the group was adopted as a typical variable.

Comparison between two groups (e.g. the patient and the control group, the old and young subjects group) was performed with Mann–Whitney test for each outcome variable in VST. Differences were reported as significant if p < 0.05.

## Results

### Relationships between Virtual Shopping Test and conventional cognitive assessment in patient group

Table 1 presents patient description and cognitive assessment data. Table 2 presents the correlation between VST and conventional cognitive assessments in patient group. In this table, we inserted the neuropsychological tests and questionnaires in which scores were correlated significantly with VST variables.

**Table 2:** Correlation between VST and conventional cognitive assessments in patient group

		**Attention**		**Memory**					
	**MMSE**	**SDMT**	**SRT**	**RBMT**					**EMC**
		**Completing rate**	**Correct rate**	**SPS**	**‘Belonging’**	**‘Appointment’**	**‘Pictures’**	**‘Date’**	
Bag use	0.06	−0.29	−0.28	−0.41	0.31	−0.5	−0.65*	0.11	0.52
List use	−0.69*	−0.5	−0.63	−0.71*	−0.67*	−0.73*	−0.34	−0.59	0.76*
Forward movement	−0.31	−0.43	−0.62	−0.34	0.09	−0.56	−0.53	−0.3	0.01
Correct purchases	0.18	0.39	0.05	0.48	0.47	0.09	0.16	0.67*	0.26
Total time	−0.6	−0.80**	−0.89**	−0.71*	−0.37	−0.88**	−0.45	−0.52	0.52

The variables on RBMT include standard profile score (SPS) and four subtests: ‘Belonging’, ‘Appointment’, ‘Pictures’, and ‘Date’.

Speaman’s correlation: *p < 0.05,**p < 0.01.

As shown in Table 2, regarding general cognitive ability, the times of List use was correlated significantly with the MMSE score on VST (r = −0.69). The significant negative correlation indicated that if the number of references to the shopping list on VST was high, then the performance of MMSE was also low.

Regarding attention, the Total time spent to complete the whole shopping task on VST were correlated significantly with the each score on two tests (the completing rate in SDMT, the correct rate in SRT) (r = −0.80 and −0.89, respectively). The significant negative correlations indicated that longer time spent to complete the whole shopping task on VST related to a poorer performance on SDMT and SRT.

With regard to memory, there were significant negative correlations between the times of List use on VST and the RBMT standard profile score and the two subtests: ‘Belonging’, ‘Appointment’ (r = −0.71, -0.67 and −0.73, respectively). There was also positive correlation between the times of List use on VST and the EMC score (r = 0.76). The significant negative/positive correlations indicated that higher number of times that the patients referred to the shopping list on VST related to a poorer performance on RMBT and more severe memory impairments on EMC. There were significant negative correlations between the times of Bag use on VST and the one subtest in RBMT: ‘Pictures’ (r = −0.65). The significant negative correlation indicated that higher number of times that the patients check the items in the bag on VST related to a poorer performance on the picture remembering task in RBMT.

There were significant negative correlations between the Total time spent to complete the whole shopping task on VST and the RBMT standard profile score and one subtest: ‘Appointment’ (r = −0.71 and −0.88, respectively). The significant negative correlation indicated that longer time was spent to complete the whole shopping task on VST related to a poorer performance on the prospective remembering task in RBMT.

There were significant positive correlations between the number of Correct purchases on VST and one subtest of the RBMT: ‘Date’ (r = 0.67). The significant positive correlation indicated that less correct items purchased on VST related to a lower orientation score in RBMT.

On the other hand, there was no significant correlation between VST variables and some conventional cognitive assessments (including Star and Letter Cancellation Task, Zoo Map Test, and DEX).

### Comparison of patient and control group performance on Virtual Shopping Test

Table 3 presents comparison of VST performance between patient and control group. There were significant differences in seven out of ten VST variables (excluding Bag use, Reverse movement, and Correct purchases) with the patient group performing worse than the control group. For example, the patient group bought correct items but they had to refer to the shopping list more often. They also moved on the street erratically and needed more time to complete the whole shopping task.

**Table 3:** Comparison of VST performance between patient and control group

**VST outcome variables**	**Patient group (n = 10)**	**Control group (n = 10)**	**p**
Bag use	0 (0–1)	0 (0–0)	0.31
List use	0 (0–3)	0 (0–0)	*
Cue use	0 (0–3)	0 (0–0)	*
Forward movement	13.5 (12–47)	12 (12–14)	*
Reverse movement	1 (0–8)	0 (0–2)	0.17
Correct purchases	4 (3–4)	4 (4–4)	0.15
Total time [s]	134 (77–605)	72 (59–107)	**
Time in shops [s]	31 (22–102)	22 (21–27)	**
Time on road [s]	99.5 (52–503)	47.5 (37–81)	**
Mean time per shop [s]	7.5 (6–19)	5.5 (5.3‒7)	**

Results show median (the minimum‒the maximum).

Mann–Whitney test: *p < 0.05, **p < 0.01.

### Comparison of old and young healthy subjects’ performance on Virtual Shopping Test

Table 4 presents comparison of VST performance between old and young group. There were significant differences in four out of ten VST variables (Total time, Time in shops, Time on road, and Mean time per shop) between the old and the young subjects groups. The old group performs worse than the young group. For example, although the old subjects bought the same number of correct items without using hints such as referring to the shopping list or looking at the shopping bag, they needed more time to complete the whole shopping task than the young subjects. There were no significant differences on Forward movement and Reverse movement between the two groups, while the ranges of these variables were wider in the old group than the young group.

**Table 4:** Comparison of VST performance between old and young healthy group

**VST outcome variables**	**Old group (n = 10)**	**Young group (n = 10)**	**p**
Bag use	0 (0–0)	0 (0–0)	―
List use	0 (0–0)	0 (0–0)	―
Cue use	0 (0–0)	0 (0–0)	―
Forward movement	12 (12–18)	12 (12–12)	0.32
Reverse movement	0 (0–6)	0 (0–2)	0.55
Correct purchases	4 (4–4)	4 (4–4)	―
Total time [s]	85.5 (68–144)	66.5 (59–83)	**
Time in shops [s]	26.5 (22–42)	21.5(20–30)	**
Time on road [s]	62 (46–112)	45.5 (37–57)	**
Mean time per shop [s]	7 (6–11)	5.4 (5.3‒7.5)	**

Results show median (the minimum‒the maximum).

Mann–Whitney test: **p < 0.01.

## Discussion

### Cognitive function related to Virtual Shopping Test

We discuss the validity of VST to assess cognitive functions of brain-damaged patients, in detail, their attention, visual perception, memory, executive function, the overall cognitive function, respectively as following.

With regard to attention, the scores on the two tests (the completing rate in SDMT, the correct rate in SRT) were correlated significantly with the Total time spent to complete the whole shopping task on VST. SDMT is closely related to divided attention, switching attention, and attention capacity [20,29]. It is also connected with the central executive system of working memory and visual sketch pad function from cognitive psychological point of view. Working memory is the function of keeping information temporarily, processing and operating it. It is also closely related with attention [30]. SRT is a visual input task related to sustained attention [21]. In this task, subjects were instructed to concentrate on a screen and they were asked to respond to the specific number as soon as possible for a specific period of time. SRT and VST both share common features to gauge attention based on visual input tasks. We considered that the function of working memory would be required when the subjects memorized the items on the shopping list while shopping.

With regard to visual perception, there was no statistically significant correlation between VST and the Star/Letter Cancellation Task. We considered that one of the reasons was the patient group did not include typical patients with USN. As shown in Table 1, the mean score of Star Cancellation Task ranged from 48 to 54 out of 54, and the mean score of Letter Cancellation Task ranged from 36 to 40 out of 40. These data indicated a ceiling effect. Further studies will be needed to elaborate on these findings.

With regard to memory, there were significant negative/positive correlations between the number of times the shopping list were used (List use) on VST and the RBMT standard profile score and the two subsets: ‘Belonging’, ‘Appointment’ and the EMC score. There was significant negative correlation between the number of times the shopping bag were used (Bag use) on VST and the one subtests in RBMT: ‘Pictures’. Similarly, there was significant negative correlation between the Total time spent to complete the whole shopping task on VST and the RBMT standard profile score and the one subset: ‘Appointment’. However, there was significant positive correlation between the number of Correct purchases on VST and the one subtest of the RBMT: ‘Date’.

RBMT has ecological validity as a set of tests for everyday memory [24,25]. The two subtests: ‘Belonging’, ‘Appointment’ are related to prospective memory, which involves bringing a previously formed plan back to consciousness at the right time and place [31]. Prospective memory is a sort of recent memory and has two characterized functions: memorizing the content and reminding it at the appropriate time [32,33]. ‘Picture’ is related to visual memory, while ‘Date’ is related to orientation. EMC is a questionnaire about everyday memory [34].

These findings reflected the fact that participants were able to encode something and retrieve the information on demand during the VST, as all these measures were related to linguistic and visual memory. One of the reasons was because participants visually memorized the items already bought on VST. It also suggested that VST could be a useful battery for prospective memory because participants were asked to memorize four shopping items and to repeatedly remind them when they approached each target shop during the performance. We considered that List use, Bag use and Total time on VST were related to everyday memory, especially prospective and visual memories.

Regarding executive function, there was no statistically significant correlation between VST and Zoo Map Test/DEX. We considered the reason was because Zoo Map Test [27] had a more difficult task with a more complex map than VST; namely it asked subjects to go to six places in a zoo. In order to prove VST could be a new test for executive function, we needed to find out its significance by comparing VST with the real performance test such as Multiple Errands Test [35]. It is performed at a real shopping mall environment and involves the completion of various tasks, rules to adhere to and a specified time frame.

In Conclusion, These results suggested that the number of times to refer to the shopping list was related to everyday memory and the general cognitive ability. The total time spent to complete the whole shopping task was related to attention and everyday memory. VST could evaluate some aspects of cognitive function on virtual shopping scene through one test session.

### The difference between brain-damaged patients and controls in Virtual Shopping Test performance

As shown in Table 3, the brain-damaged patient group performed significantly worse than the control group on seven out of ten VST variables: List use, Cue use, Forward movement, Total time, Time in shops, Time on road, and Mean time per shop. The significant difference between the patient and control groups in time required to complete the task was consistent with the findings of previous study using Virtual Mall in patients with stroke [36]. Similarly, the significant difference between the two groups in the scores related to event-based prospective memory was consistent with the findings of previous study using Virtual Library Task in patients with TBI [37].

We believe that VST is applicable to patients with cognitive dysfunction without severe aphasia and/or severe USN. We consider that not only the number of items bought correctly, but also the times referring to hints, the times of movement, and the total time spent on road/shop are important points for cognitive assessment of the brain-damaged people using VST.

### The basic age difference in Virtual Shopping Test performance

As shown in Table 4, the old group performed significantly worse than the young group on four out of ten VST variables: Total time, Time in shops, Time on road, and Mean time per shop. It was suggested that the longer time required in the old subjects group in VST was related to the decline in their cognitive process and their ability in physical movement. However, there was no statistical difference between the two groups on other variables: Bag use, List use, Cue use, Forward movement, Reverse movement, and Correct purchases.

The significant difference between the old and young group in not behavioral variables such as the number of correct answer but the total time spent to complete the task was contrary to the findings of previous study using CPT. In the study, it was suggested that increasing age was associated with increased numbers of commission and false alarm errors, while older subjects were not significantly slower at responding to stimuli than younger subjects [38]. We considered the reason was because subjects tried to complete the whole shopping tasks correctly on VST even if it took longer, which was similar to the way they behave in their daily life.

Therefore, we will need to take the age-related performance, especially the time required into consideration on VST. In order to standardize VST for a wide clinical application, we will need to collect data and establish the nominal value for each age group in VST. Results from VST in the clinic may also underestimate the practical cognitive problems experienced by old people with mild cognitive impairments.

## Conclusions

We have developed a shopping test with the use of a virtual reality system for assessing cognitive function. This study investigated its significance by comparing the Virtual Shopping Test (VST) performance with the results of other neuropsychological tests and questionnaires. VST was also applied to brain-damaged patients and the basic age differences in VST performance were studied.

As a result, some VST variables correlated with the scores of other cognitive assessments related to attention and everyday memory. The results demonstrated that VST could be used to evaluate the ability of attention and memory in patients with brain damage through one test session.

In addition, the mean number of times that subjects refer to the shopping list, the mean number of subjects' movements, and the mean total time to complete the task were all significantly larger/longer for the patients with brain damaged than for the subjects in the control experiment. The mean total time for completing the VST was also significantly longer for the old subjects than for the young subjects. Therefore, we concluded that VST can be used as a cognitive assessment tool in rehabilitation for brain-damaged patients.

## Abbreviations

ADL: Activities of daily living; Bag use: the number of times to check the items bought in the bag; BIT: Behavioural Inattention Test; Correct purchases: The number of items bought correctly; CPT: Continuous Performance Test; Cue use: the total number of times to check the shopping list and bag; DEX: Dysexecutive questionnaire; EMC: Everyday memory checklist; Forward movement: the times to press upper arrow; IADL: Instrumental activities of daily living; List use: the number of times to check the shopping list; Mean time per shop: the average time spent in one shop; MMSE: Mini-mental state examination; RBMT: Rivermead behavioural memory test; Reverse movement: the times to press lower arrow; SDMT: Symbol digit modalities test; SRT: Simple reaction time task; TBI: Traumatic brain injury; Time in shops: the total time spent in each shop; Time on road: the total time spent on road; Total time: the total time spent to complete the whole shopping; USN: unilateral spatial neglect; VR: Virtual reality; VST: The vrtual shopping test.

## Competing interests

The authors declare that they have no conflicts of interests financially in this research.

## Authors' contributions

SO designed VST, collected/analyzed data and wrote the manuscript. KS and AN provided feedback and expert guidance throughout this study. ZL provided feedback and expert guidance throughout this study, and contributed to the intellectual content of this manuscript. MK participated in the design of the VST. TF participated in the revisions of the manuscript. All authors read and approved the final manuscript.
